# Days of Future Passed: Best Calibration Does Not Guarantee Reliable Forecasts of Future Tick Distributions

**DOI:** 10.3390/pathogens15080863

**Published:** 2026-08-19

**Authors:** Agustín Estrada-Peña

**Affiliations:** Ministry of Human Health, FCSAI Foundation, 28029 Madrid, Spain; antricola@me.com

**Keywords:** ticks, species distribution models, maxent, boosted regression trees, climate change, transferability, Boyce index, ecological niche, vector surveillance, *Ixodes*, *Amblyomma*, *Dermacentor*

## Abstract

Forecasting species distributions under future climate scenarios has become a standard application of Species Distribution Models (SDMs), yet their temporal transferability is rarely evaluated. We assessed the reliability of future projections using four widespread tick species (*Amblyomma americanum*, *Dermacentor variabilis*, *Ixodes ricinus*, and *Ixodes scapularis*) with occurrence records spanning from 1961–2025. Models were calibrated sequentially using decade-specific records and projected onto subsequent decades, for which independent observations were available. Climate predictors derived from TerraClimate using harmonic regression were modelled with Boosted Regression Trees (BRT) and Maxent. Transferability was evaluated using the Boyce Index, Schoener’s *D*, and niche dynamics. Both algorithms achieved satisfactory calibration, but temporal projections frequently failed to reproduce the climatic niche represented by independent records. Although BRT consistently outperformed Maxent, forecasting performance depended primarily on the proportion of the realised climatic niche represented during calibration rather than on the number of occurrence records. Models with excellent calibration statistics often showed poor temporal transferability, whereas models trained on progressively more complete niche representations produced substantially more reliable forecasts. Our results demonstrate that reliable model calibration does not guarantee reliable forecasts of future tick distributions, suggesting that incomplete niche representation acts as a major constraint on long-term SDM projections under the evaluated framework.

## 1. Introduction

Research into ticks and their transmitted pathogens has flourished in recent decades, driven by the need to predict how vector populations respond to local or global climate and changes in land use and land patterns [1]. Early research focused strictly on climate, but studies have since integrated landscape, land use, and biotic interactions. To mitigate the future impact of tick-borne diseases, understanding how climate change will alter vector and host distributions is critical [2].

Understanding how climate change may affect future tick distributions is essential for anticipating risks to animal and human health [3]. Species Distribution Models (SDMs) have emerged as one of the most widely employed tools for understanding the likely distribution of ticks and the pathogens they transmit [4], with the aim of implementing prevention measures in human and animal health. These models integrate occurrence records with climatic and environmental predictors to estimate habitat suitability under both current and future conditions. Species Distribution Models have exploded in popularity applied to the field of ticks: a query in Scopus conducted at the inception of this study (ca. November 2025) showed that nearly 5000 papers were published on tick SDMs between 2000 and 2025, with roughly 35–40% (1100–1400 papers) forecasting distributions under future IPCC climate scenarios (RCPs/SSPs).

Although the introduction of environmental modeling to ticks is a relatively recent field, the methodology has been fed with numerous and different strategies [5,6], including, but not limited to, different algorithms aimed to detect patterns of presence/absence, pseudo-absences, or background points. All of them represent different concepts that are commonly confused in most papers (as explained by [7]). Pseudo-absences are locations where the species was not recorded and are treated as true absences in the model. Most commonly, only presence data are available, and pseudo-absences provide a contrast against which the model can learn what niche the species does not occupy. Background points are randomly collected points across a territory, and they make no claim about absence. The model learns to contrast the explanatory variables in the presence locations against this background [8].

Among the available modeling approaches, the algorithm Maxent [9] gained rapid favor due to its predictive strength, flexible mathematical framework, free availability, and accessible implementation [10,11]. Combined with WorldClim variables, it is by far the most frequently used framework for projecting future tick distributions [5,6]. However, transferring SDMs across time and space presents well-known methodological challenges. WorldClim variables are often highly collinear, and the strength of these correlations may change over time as the climate evolves [12]. Although variable selection procedures based on variance inflation factors (VIF) are commonly recommended, removing variables solely on statistical grounds may exclude predictors that are ecologically relevant for the target species, potentially biasing model calibration and future projections [13,14,15].

Boosted Regression Trees (BRT) represent another widely used approach that selects hundreds or thousands of regression trees in a forward stage-wise fashion. At each step of model fitting, the algorithm focuses on the weakest parts of the model built so far (the observations that so far are not predicted accurately) by fitting each new tree to the residuals of the previously fitted trees [11].

Maxent remains the predominant algorithm for projections of SDMs into a future environment. However, its transferability to novel temporal scenarios requires careful parameterization and training. A major concern across climate-driven SDMs is the temporal instability of collinearity within the WorldClim dataset, the primary climate source for tick SDMs [6]. As climate shifts, historical correlations between variables change [10,11,16]. However, validating these long-term predictions (50–100 years) is challenging due to uncertainties regarding future climate trajectories [16], biotic adaptation, migration velocity, spatial scale effects, extinction rates, and population dynamics [17,18].

While Maxent’s L1 regularization (LASSO-type penalty) mitigates collinearity during training by driving redundant coefficients to zero, projecting any model under altered correlation structures remains complex. During training, a model with highly correlated variables can still yield a high Area Under the Curve (AUC) [19]. A key challenge during temporal projections arises from “collinearity shifts” [20]. For instance, asymmetric climate warming can break historical correlations between winter and annual temperatures, creating novel environmental combinations. If an algorithm relies heavily on specific variable combinations that uncouple over time, extrapolation can become uncertain, potentially leading to niche overestimations or underestimations in biologically critical areas.

Consequently, standard protocols dictate that the full set of Bioclim variables should never be used without prior filtering via VIF [17] or Principal Component Analysis (PCA). However, this transformation removes the direct ecological interpretation of individual predictors. PCA, for instance, tends to “blend” the effects of particular details of climate phenology that are critically important for tick survival and development. In both cases, variable selection thresholds (e.g., VIF cutoffs without direct biological significance) may lead to a mathematically robust model that loses critical ecological sensitivity when projected into unexpected climate combinations. This represents a critical challenge in SDM modeling of ticks when projected into future conditions.

This study evaluates the temporal transferability of climate-driven SDMs using four widespread tick species, a common set of climatic predictors, and two modeling algorithms (Maxent and BRT). Tick occurrence records with known collection dates, spanning from 1961 to 2025, were used to calibrate models for successive decades and project them onto subsequent periods for which both climate data and independent occurrence records were available. This unique framework enables direct evaluation of forecasting performance by comparing projected suitability with observed distributions, rather than relying on hypothetical future scenarios. By combining measures of model calibration, temporal transferability, and niche similarity, we examine the factors determining the reliability of long-term projections. We hypothesised that forecasting performance depends primarily on the completeness with which the realised climatic niche is represented in the calibration data, rather than on the absolute number of occurrence records available. Consequently, we predict that models trained on increasingly representative portions of the climatic niche will exhibit greater temporal transferability, irrespective of the modeling algorithm employed.

## 2. Materials and Methods

### 2.1. Dataset of Records

**Figure 1:**
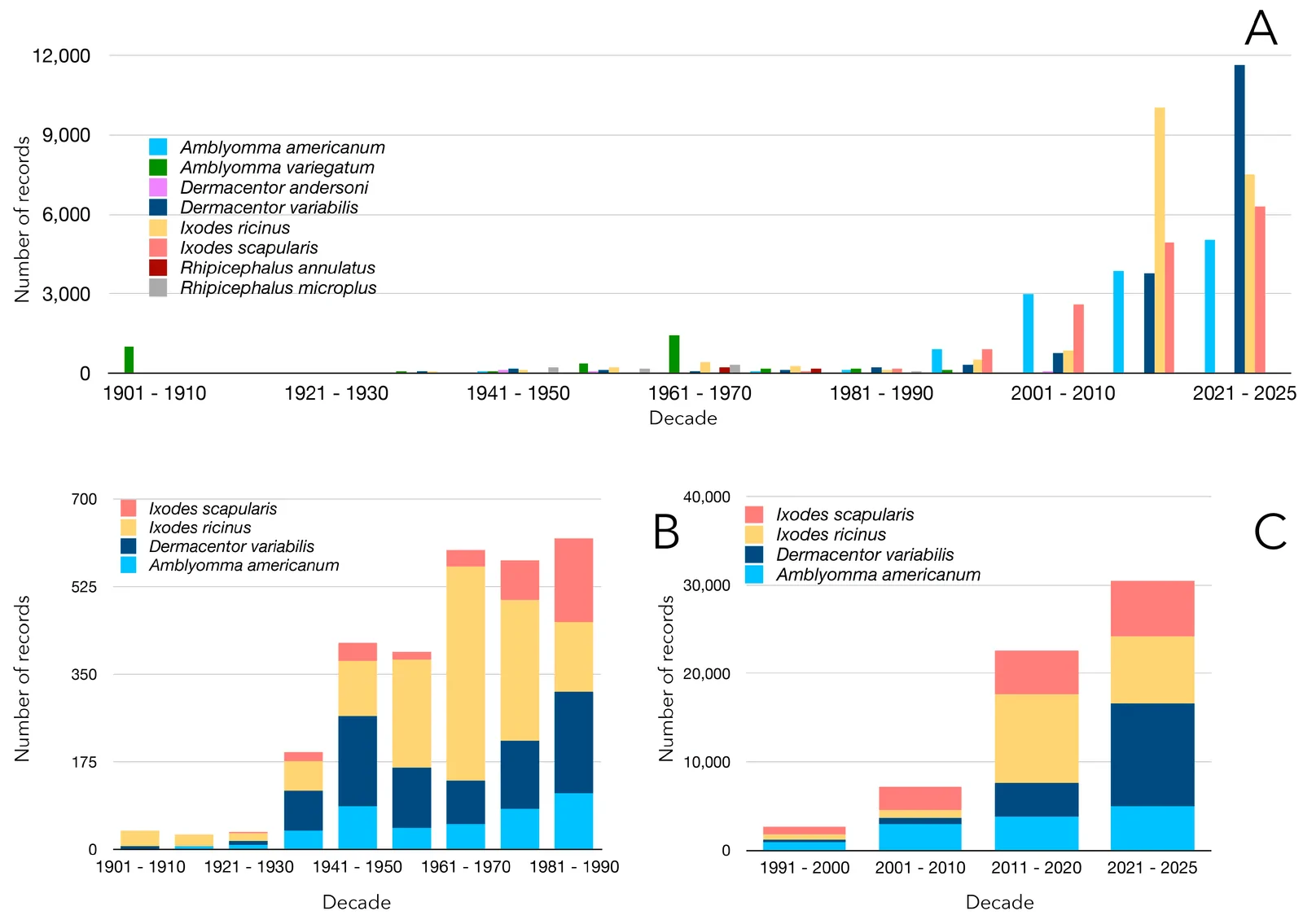
The records of ticks with coordinates and the year of collection, as available in digitally available sources. (**A**) The total number of records of each species of ticks initially considered for this study and collected at each period of 10 years (the last period includes only 6 years, from 2020 to 2025). (**B**) The trend in time of the records of the four species of ticks finally included in the study, in the period 1901–1990. (**C**) The trend in time of the records of the four species of ticks finally included in the study, in the period 1991–2025.

Eight species were originally included in the query for this study. Notably, we recorded few dated and georeferenced records of four species collected after the year 2000, which would prevent the training and projection of the models for these species and the same pace as the others. These four species were *A. variegatum*, *D. andersoni*, *R. annulatus,* and *R. microplus*. On the other hand, both *R. annulatus* and *R. microplus* are known to behave as invasive species. These two species are reported as new permanent foci or re-colonized areas that were previously cleaned. This could lead to the anomalous detection of their core habitat and have an impact on the training and projection of models. We thus removed all the records of *A. variegatum*, *R. annulatus*, and *R. microplus* in subsequent analyses. We used dated (year) and georeferenced records of *Ixodes ricinus*, *Ixodes scapularis*, *Amblyomma americanum*, and *Dermacentor variabilis*. All the tick records were obtained from the previous compilations [1,21]. The total of records for each tick species and date of collection (decade) is included in Table 1 and Figure 1.

### 2.2. Climate Data

Climate data used to model environmental suitability for ticks were obtained from the Terra-Climate repository (https://www.climatologylab.org/terraclimate.html, accessed on 18 August 2026, [22]). Monthly data for maximum temperature (TMAX), minimum temperature (TMIN), and vapour pressure deficit (VPD) were compiled for the period from 1961–2025, at monthly intervals, for the complete World. Data were divided into 10 years periods, and we included the last period (2021–2025), which spanned only 5 years.

Harmonic regression [23] was performed on the monthly averages of 10 years, independently for each variable; the first three coefficients of each regression were retained as explanatory variables, representing average, amplitude, and phase. This yielded nine climatic predictors: three coefficients each for TMAX, TMIN, and VPD. All the calculations were done in R (v. 4.0.2) [24], using the package terra (v 1.8–60 [25]). The script used to calculate harmonic regression was provided [26].

We did not include other data commonly used in species distribution modeling, like the “bio” set of variables of WorldClim. The purpose is to compare the projection of trained models at different chunks of time and projects into different time slices. This is unavailable in that set of WorldClim-derived variables. The purpose of this study is not comparative performance of climate datasets, but the unreliability of models projected into the future with our current imperfect knowledge of tick distribution, and thus the skewed perception of the tick environmental niche.

### 2.3. Modeling

We generated maps of the potential niche of the target species, spanning from 1961–1970, using records with the annotated decade of collection, with a resolution of 4 km. We adhered a strategy based on the production of the best model with records of a decade and its projection to the next ones. We used two algorithms: (a) Maxent [27], implemented in R as “maxnet” v 0.1.4, and a BRT implemented in R as the library “gbm” v.2.2.2 [28]. Maxent and boosted regression trees are still among the top performing models [11]. This algorithm approach was chosen to robustly capture different dimensions of the ecological response of the ticks because Maxent is known to be sensitive to collinearity, but BRT is not [6]. Maxent may also show issues of projection because the collinearity of the variables, to which BRT remains stable. We wanted to know if a widely used algorithm produced similar projections than another robust approach like BRT since some of the raster explanatory layers have a relatively high degree of collinearity.

Maxent was deployed using a complementary log–log (cloglog) link function to generate continuous habitat suitability surfaces ranging from 0 to 1. We optimised the parameters of feature classes and the regularization parameters, as recommended. To select the best model parameters, the feature classes were restricted to linear, quadratic, and product. The regularization multiplier values were 1, 2, and 5. Parameters not specified were kept at their default values in the package. Boosted Regression Trees (BRT) were used with the library gbm. For running the algorithm, we used a tree complexity = 5, learning rate = 0.005, and bag fraction = 0.75.

The strategy is aimed to train models with the climate of every decade and project to the following decades, checking the accuracy of the projections. To guarantee that the models’ predictive power was assessed with rigor, evaluation metrics were calculated across both continuous and binarized outputs. Continuous suitability maps were converted into binary presence–absence predictions by applying an optimal threshold criterion that maximized the True Skill Statistic (TSS). This threshold-selection method balances omission and commission errors evenly, preventing the artificial inflation of suitability in poorly sampled regions. We considered several metrics: sensitivity (the proportion of correctly predicted presences), specificity (the proportion of correctly predicted absences), and threshold (maximizing both specificity and sensitivity).

### 2.4. Model Evaluation and Statistical Analysis

The critical test to evaluate the ability of each model to predict the “future decades” was the Boyce index, coming from the previous evaluation according to [7]. Model performance was evaluated using the Boyce index, a presence-only metric suitable for species distribution and habitat suitability models. This index measures the extent to which model predictions differ from a random distribution of observed presences by partitioning continuous suitability predictions into intervals (or moving windows) and calculating the ratio of predicted to expected presences.

The Boyce index ranges continuously from −1 to +1: positive values indicate consistent model predictions where presence probability increases with suitability; a value of 0 reflects performance no better than random chance; and negative values indicate an incorrect or inverse model that predicts low suitability where species presence is high. In short, Boyce index measures the transferability of the model to another set of conditions. A high value in the Boyce index (near 1) indicates that the model trained with climate and records of a given decade can describe the climate and records of the decade of projection. As the suitability for the tick increases, more records are captured, and there is a lineal relationship between the suitability and the number of records; this lineal correlation is balanced by the background values: as the suitability decreases, more background points are captured.

To mathematically compute the Boyce index, the continuous model predictions ranging from 0 to 1 were partitioned into discrete intervals (m bins). For each bin i, a predicted-to-expected ratio ***r_i_*** was calculated as

$$r_{i}=\frac{\frac{n_{1i}}{n_{1}}}{\frac{n_{0i}}{n_{0}}}$$

where ***n***_1*i*_ and ***n***_1_ represent the validation presences in bin *i* and total presences, respectively, while ***n***_0*i*_ and ***n***_0_ correspond to the background points within bin *i* and across the entire study area. The final Boyce index was derived by calculating the Spearman rank correlation coefficient between the bin suitability ranking and their corresponding ***r****_i_* values.

To evaluate ecological niche dynamics and shifts between the baseline (training) and projected periods, we applied the centering, calibration, and niche equivalency framework already proposed [29] using the ecospat R package [30]. First, a global PCA was calibrated using a unified environmental background dataset consisting of random spatial samples (n = 5000 per decade) from both the training and projection areas. Then, values of niche stability, niche, and niche unfilling were calculated from the data above. High stability (close to 1) means that the geographic areas the model identified as suitable in the past remain suitable in the future, and that the ideal niche stays in the same physical location. Low stability (close to 0) means that the suitable areas of the past cease to be suitable in the future, and the niche is “moving” to another location (which will be reflected in an increase of expansion or unfilling). Expansion (in the range 0 to 1) means new areas colonised by a tick in the simulation. Unfilling is the contrary term, in which portions of the niche previously colonised by the tick (trained model) have low suitability in the “future” (projected model).

To assess the temporal transferability of the Maxent ecological niche models, an iterative temporal cross-validation framework was implemented across all decade combinations (from 1961–1970 to 2021–2025). For each species, models trained on the climate conditions and occurrence records of a specific decade (D_source_) were projected onto the environmental scenarios of all subsequent chronological decades (D_target_). The aim is to simulate the projection of a niche into future climate conditions, for which actual records exist and for which an explicit assessment of performance can be examined for each combination of species/decade/algorithm. The predictive performance and niche similarity of these hindcasts/forecasts were quantified using two metrics: Schoener’s *D* and continuous Boyce index.

Schoener’s D measures spatial niche overlap and assess potential model decay over time. This metric compared, on a pixel-by-pixel basis, the suitability surface predicted by the temporal transfer (D_source_ → D_target_) against the baseline contemporary model trained directly within that same target period (D_target_ baseline). The index ranges from 0 (no overlap) to 1 (identical niche predictions) [31].

The index was calculated at the pixel level using the following equation:

$$D\left(p_{eval},p_{ref}\right)=1-\frac{1}{2}\sum_{i=1}^{N}\left|p_{eval,i}-p_{ref,i}\right|$$

where $p_{eval,i}$ and $p_{eval,i}$ represent the normalized suitability values (acting as probability densities) at pixel i for the evaluated decade and the reference decade, respectively, and N is the total number of pixels in the study area.

The Continuous Boyce Index (CBI) was employed to evaluate the predictive accuracy of the transferred models. For each decade pair (D_source_ → D_target_), the CBI was computed by extracting the projected suitability values at the independent presence coordinates recorded exclusively during the D_target_ period. CBI values range from −1 to +1, where values close to +1 indicate high predictive performance consistent with future observed distributions.

We assumed two different perspectives, namely (a) the records of one decade model, the consecutive one, and the niche overlap are calculated between these consecutive periods; (b) records of each decade accumulate and predict the consecutive decade period. In the first case, we want to know how the niche gained in a decade is able to be project in the consecutive one (short period of time); in the second, we want to simulate the accumulation of niche captured by decades of records to demonstrate a correlation between Boyce index and the niche similarity.

To formally evaluate the statistical strength of the relationship between historical niche overlap (measured via Schoener’s D) and projection transferability (measured via the Continuous Boyce Index), ordinary least-squares (OLS) linear regressions were fitted for each species across temporal and spatial transfers. The slope (m), coefficient of determination ($R^{2}$), and associated p-values were calculated to test whether niche completeness significantly predicted transferability success.

## 3. Results

### 3.1. Data Description and Record Distribution

**Figure 2:**
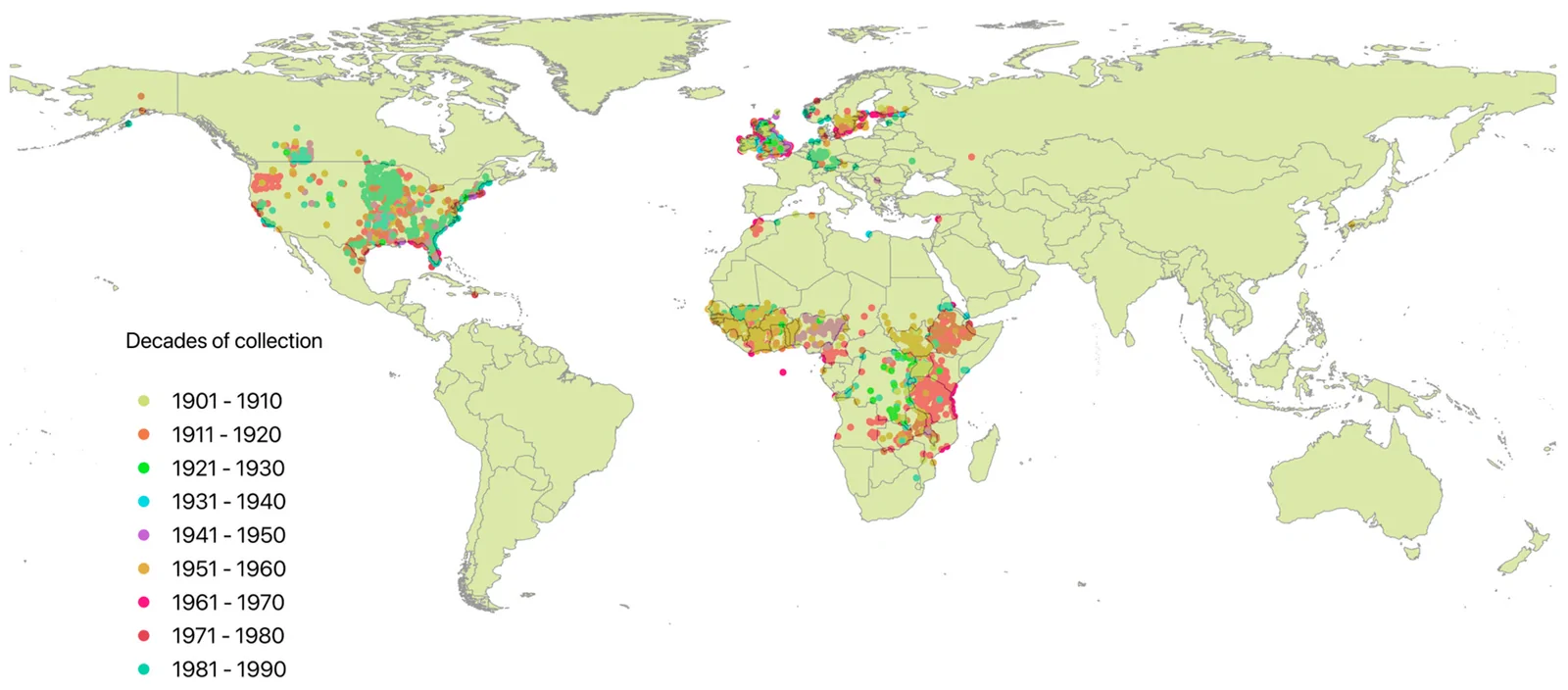
The records of ticks collected between the years 1901 and 1990. The chart is not intended to track the colours marking each decade of collections, but to show the large gaps existing in the known distribution of ticks. Notably, most of Europe lacks geo-referenced records of *I. ricinus*; the range of *I. scapularis* was not completely captured in large portions of the USA, in parts that are known to have colonised by the tick. However, *A. variegatum* was already well mapped in Africa. This map was prepared with open data for country limits available at https://www.naturalearthdata.com, and then overlayed with points using qGIS (www.qgis.org, accessed on 12 December 2025).

**Figure 3:**
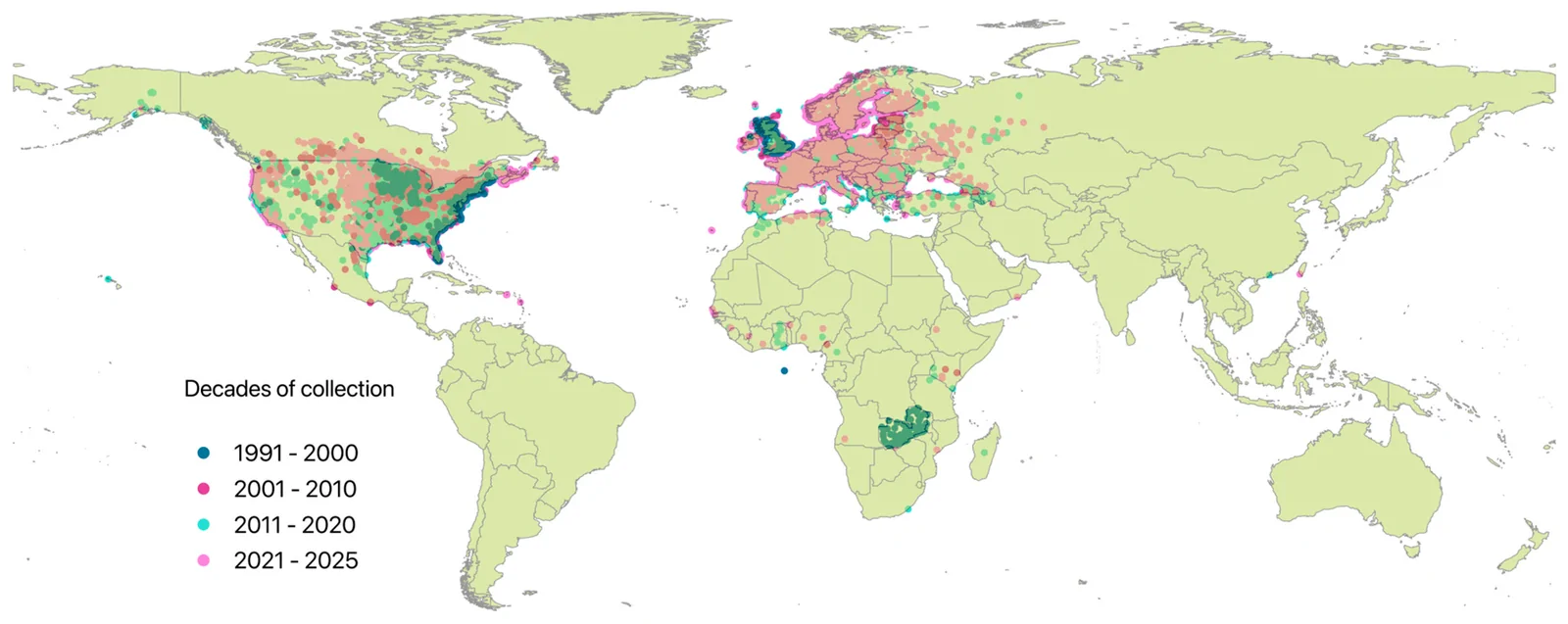
The records of ticks collected between the years 1991 and 2025. The chart is not intended to track the colours of each decade of collections, but to show the extraordinary increase in tick awareness that leaded to extensive collections in Northern Hemisphere. In example, almost the complete Europe is covered by records, as well as a large portions of the USA. However, the knowledge of the actual distribution of *A. variegatum* in Africa, including geo-referenced records, had not even minor progress, but for one country. This map was prepared with open data for country limits available at https://www.naturalearthdata.com, and then overlayed with points using qGIS (www.qgis.org, accessed on 12 December 2025).

We included four species of ticks in this study, namely *A. americanum*, *D. variabilis*, *I. ricinus*, and *I. scapularis*, for which we compiled 65,868 unique georeferenced records with the year of collection. Four species were removed from the original compilation, namely *D. andersoni*, *A. variegatum*, *R. annulatus*, and *R. microplus*, because of a low number of records or a sudden lack of reported records at certain time intervals and sharp fluctuations decades (e.g., *D. andersoni* and *A. variegatum*). Tropical species were reported more frequently in decades prior to 1990 but remain unreported with georeferenced after that year. Notably, the number of georeferenced and dated records for the eight species originally considered in this study during the 1901–1990 period was 7070 (Figure 2), whereas 63,405 records have accumulated since 1991 (Figure 3). The remaining species exhibited a steady increase in records over time, with the sharpest acceleration occurring after 1990. Overall, there is a pronounced temporal imbalance in the availability of georeferenced records across the target dates. The initial models were thus trained with climate data from the decade 1961–1970.

### 3.2. Algorithm Performance and Temporal Transferability

**Figure 4:**
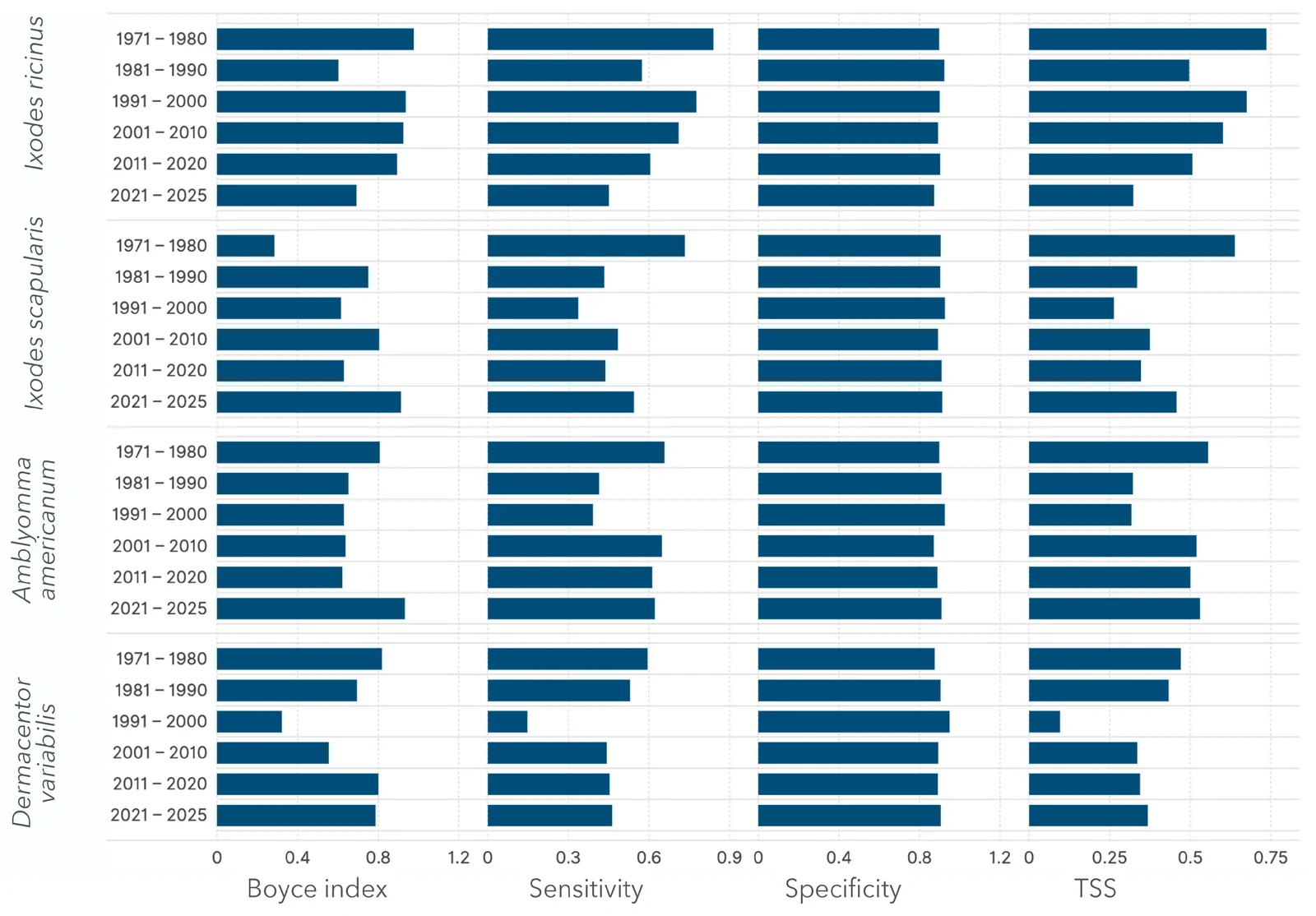
Values of the Boyce index, Sensitivity, Specificity, and the True Skill Statistic, for each species of ticks and with models trained by Boosted Regression Trees. Models for a given decade were trained with the climate and the records of each species in that decade, and projected into the future in intervals of 1, 2, 3, 4, or 5 decades (i.e., projected with the climate of the “future” decade). Models for 1971–1980 were trained only with data from 1961–1970. Models for 2021–2025 were trained with data from 1961–1970 to 2011–2020, and the results are the average of each decadal projection.

Models trained with either of the two algorithms produced similar average results when projected onto consecutive decades. Figure 4 shows the averaged values of the Boyce index, sensitivity, specificity, and TSS for models trained and projected with BRT. Each bar includes the performance index values averaged across all training models. For example, the values for the decade 2001–2010 include the average of models trained with records and climate data from the periods 1961–1970 to 1991–2000 and projected onto the consecutive decade.

No single tick species consistently yielded superior outcomes across all scenarios. Specificity was consistently high, while sensitivity displayed medium values; therefore, the niche was adequately captured in the training decade. However, the Boyce index occasionally performed poorly at random, sometimes in older decades, sometimes in recent ones, denoting an issue with transferability. The straightforward implication is that the environmental niche captured during the decade of training was poor at explaining the niche in the decade of projection.

**Figure 5:**
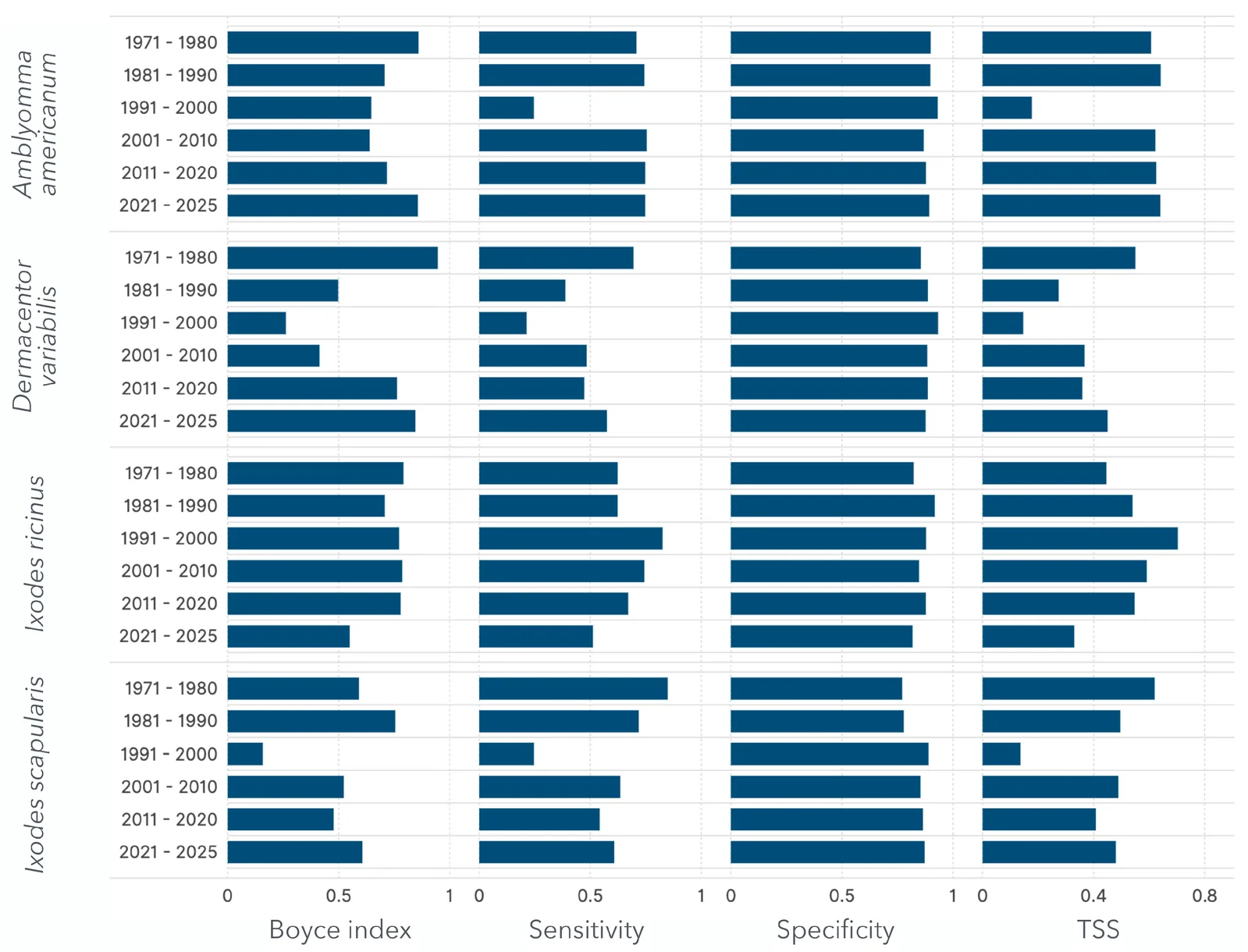
Values of the Boyce index, Sensitivity, Specificity, and the True Skill Statistic for each species of ticks and with models trained by Maxent. Models for a given decade were trained with the climate and the records of each species in that decade and projected into the future in intervals of 1, 2, 3, 4, or 5 decades (i.e., projected with the climate of the “future” decade). Models for 1971–1980 were trained only with data from 1961–1970. Models for 2021–2025 were trained with data from 1961–1970 to 2011–2020, and the results are the average of each decadal projection.

Results from Maxent (Figure 5) were not different from those built on the BRT algorithm. Certain species and algorithms exhibited unexpected performance peaks; for instance, intermediate training windows occasionally produced better models than those relying on the most recent decade of tick surveys.

**Figure 6:**
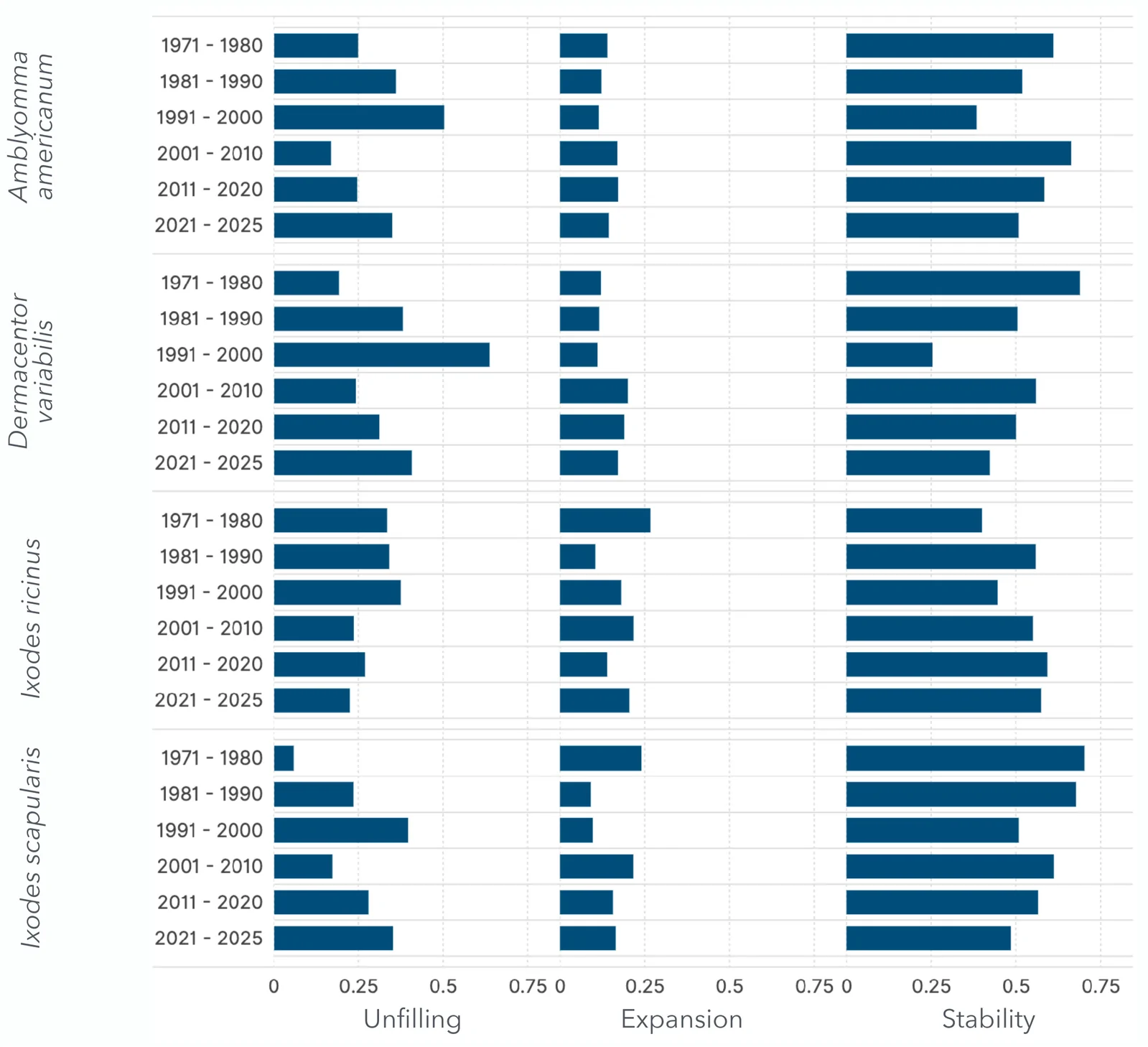
The measures of the environmental niche of four tick species, including the unfilling, the expansion, and the stability, with models trained with Boosted Regression Trees. Models are trained with the climate and the records of each species in that decade, and projected into the future in intervals of 1, 2, 3, 4, or 5 decades (i.e., projected with the climate of the “future” decade). Models for 1971–1980 were trained only with data from 1961–1970. Models for 2021–2025 were trained with data from 1961–1970 to 2011–2020, and the results are the average of each decadal projection.

**Figure 7:**
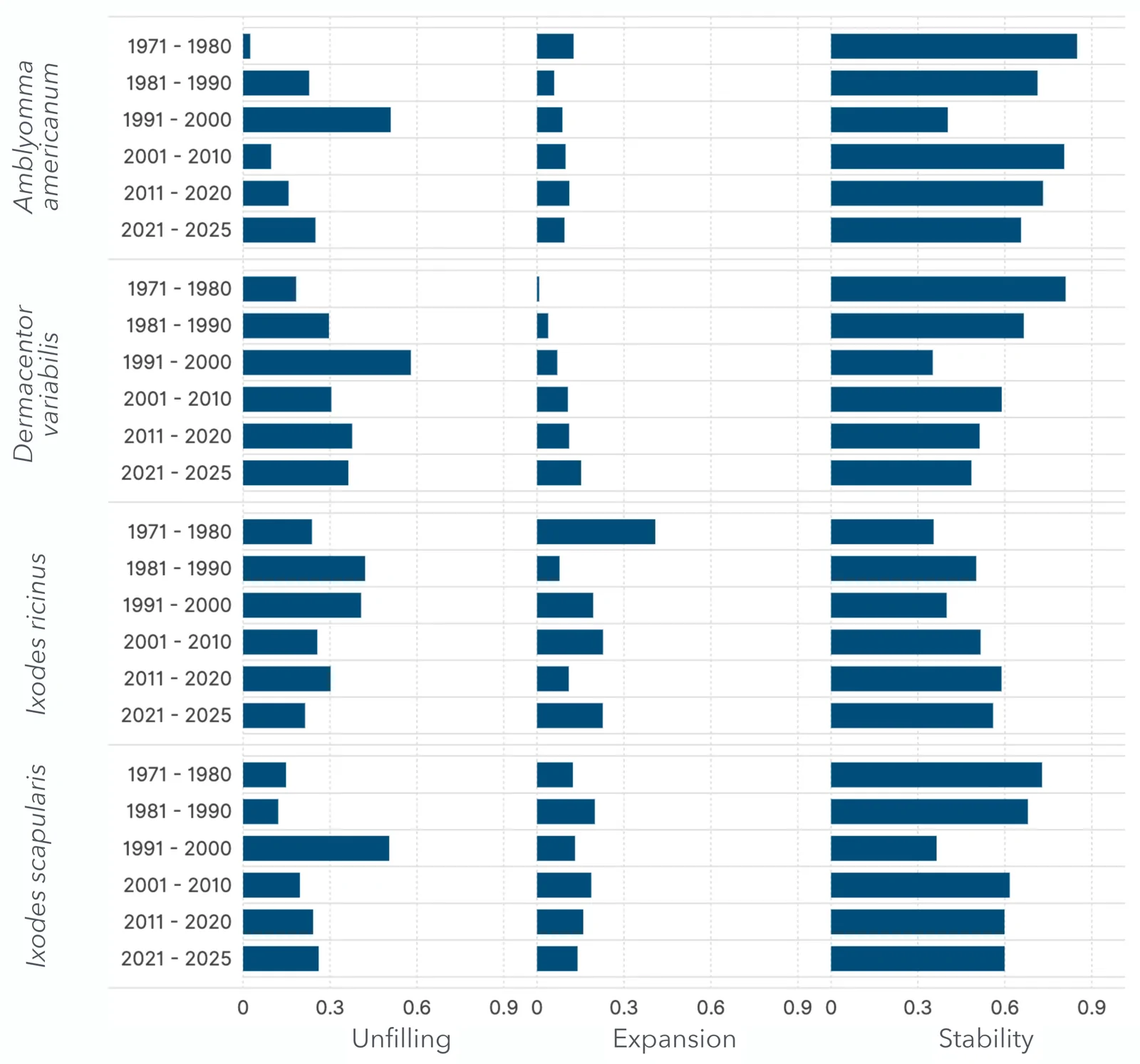
The measures of the environmental niche of four tick species, including the unfilling, the expansion, and the stability, with models trained with Maxent. Models are trained with the climate and the records of each species in that decade and projected into the future in intervals of 1, 2, 3, 4, or 5 decades (i.e., projected with the climate of the “future” decade). Models for 1971–1980 were trained only with data from 1961–1970. Models for 2021–2025 were trained with data from 1961–1970 to 2011–2020, and the results are the average of each decadal projection.

The performance indices related to the definition of the niche by the models tell a different story in Figure 6 (BRT) and Figure 7 (Maxent). The stability of the niches is relatively high (up to 75%) in models projected onto data from the initial decades. However, stability is lower (around 50%) in models projected into the most recent decades. Low values of stability are mirrored by high values of expansion, ranging between 10% and 25% of “new habitat”, and high values of unfilling, which exceed 25%. Data from the models trained with the same set of data and explanatory variables using Maxent yielded slightly poorer results (Figure 6), in which values of unfilling and expansion were higher, and niche stability slightly decreased compared to the previous cases.

In both sets of algorithms, the performance of data projections from one given decade into future time slices is relatively good, but the values of indices supporting the efficacy of capturing the niche are low. We interpret these results as unstable model projections into future decades: the algorithm cannot fully capture the tick’s niche, and, when trying to predict the niche in a “future” climate, it relies on incomplete information. The outcome consists of low values of stability (the niche changes by up to 50% when comparing training and projection), high expansion without a defined pattern (the algorithms output an increase in the niche available for the tick), and high unfilling (the “original” niche of the tick during training is not available in the projection). The expansion or unfilling indices should not be understood as spatial measures reflecting the geographic size of the tick’s range, but rather as portions of the environmental niche.

### 3.3. Niche Overlap and Cumulative Data Effects

We aimed to compare the transferability of the models when data on tick records and climate are cumulatively included in the training dataset (1, 2, …, 6 decades) and projected onto different periods. As measures of performance, we compared the Boyce index with Schoener’s *D*, the first estimating transferability between model scenarios, and the second calculating the amount of niche overlap across different decades. The aim is to demonstrate that there is a direct relationship between both indices, and that the greater the niche similarity between decades, the better the transferability of the model.

**Figure 8:**
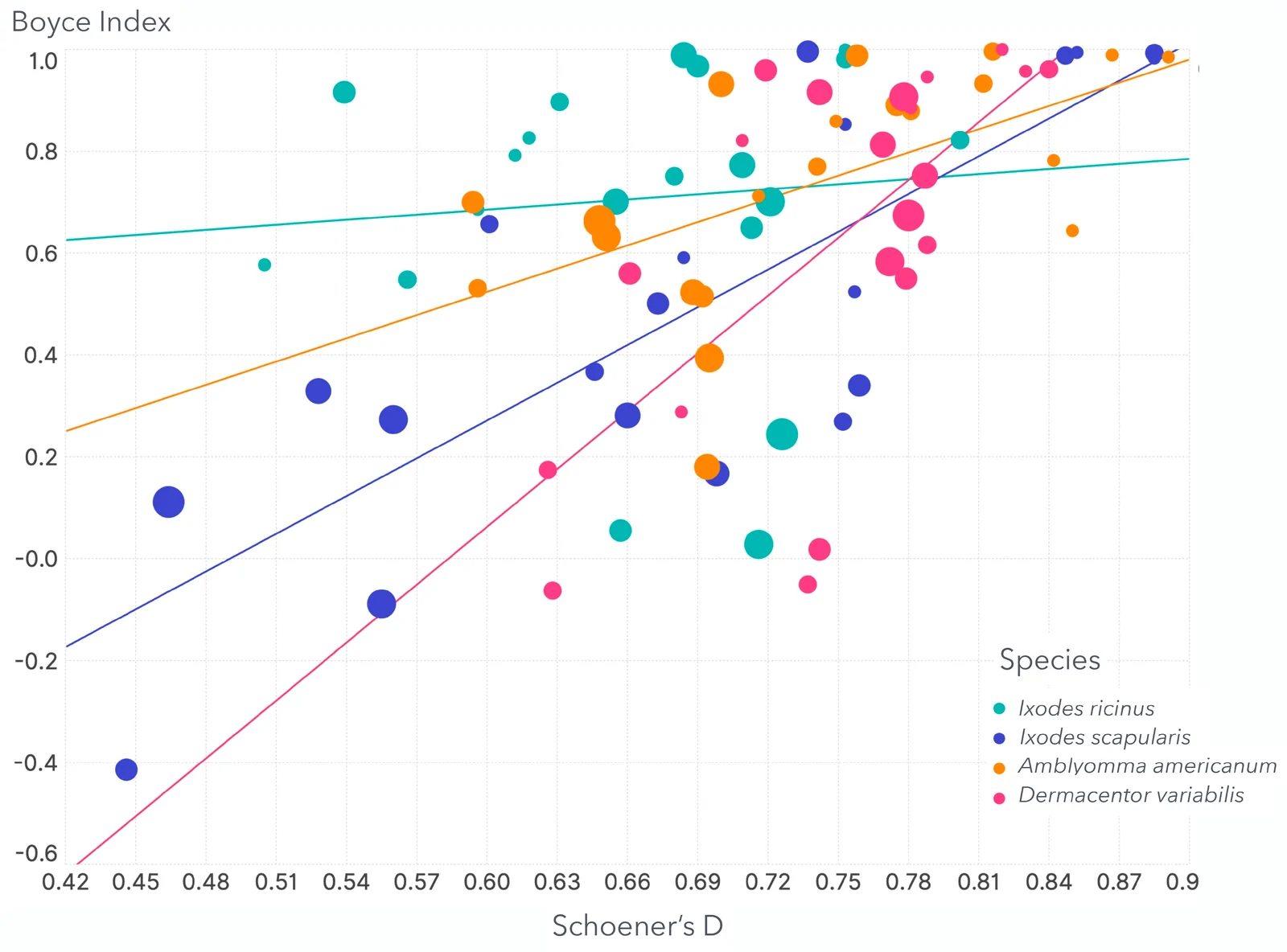
The relationships between niche overlap and Boyce index, for each species of tick, using Boosted Regression Trees. The chart represents the transferability values (Boyce index) and the niche overlap (Schoener’s *D*) obtained in models trained with the tick records reported in a decade, using the climate variables of that decade, then projected to the climate of the consecutive decade. The colours of the dots and the labels refer to the species, and the size means the number of models for each decade (minimum = 1; maximum = 6). Regression lines are included, separately for each species. Values for regression lines are (slope, R^2^, all have *p*-value = 0.001): *Ixodes ricinus*: 3.78, 0.41; *Ixodes scapularis*: 2.47, 0.68; *Amblyomma americanum*: 1.51, 0.34; *Dermacentor variabilis*: 0.33, 0.08.

**Figure 9:**
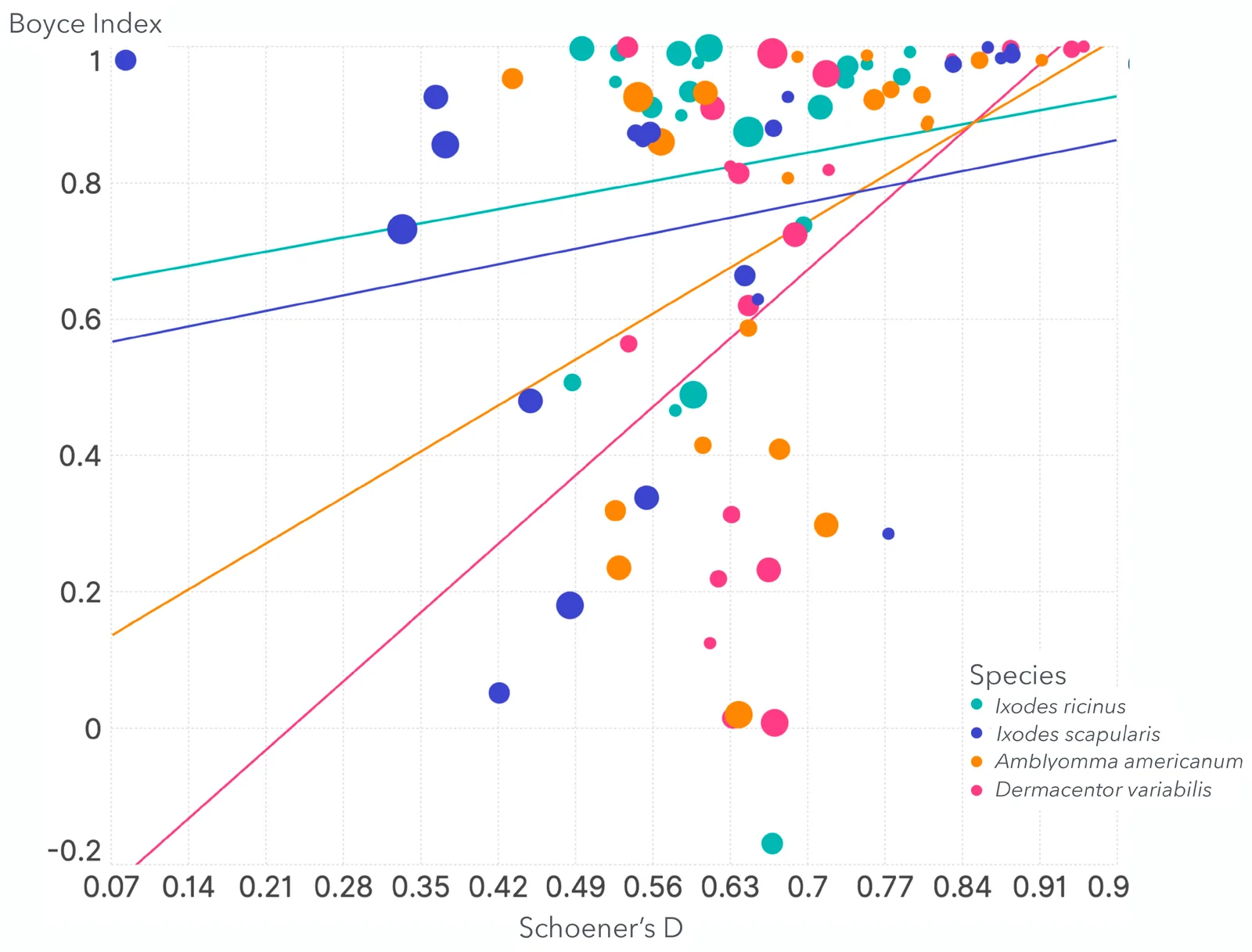
The relationships between niche overlap and Boyce index, for each species of tick, using Maxent. The chart represents the transferability values (Boyce index) and the niche overlap (Schoener’s *D*) obtained in models trained with the tick records reported in a decade, using the climate variables of that decade, then projected to the climate of the consecutive decade. The colours of the dots and the labels refer to the species, and the size means the number of models for each decade (minimum = 1; maximum = 6). Regression lines are included separately for each species. Values for regression lines are (slope, R^2^, all have *p*-value = 0.001): *Ixodes ricinus*: 3.19, 0.44; *Ixodes scapularis*: 1.19, 0.61; *Amblyomma americanum*: 2.18, 0.61; *Dermacentor variabilis*: 0.31, 0.01.

A clear relationship was observed between the fraction of the niche captured by existing records (Schoener’s *D*) and the Boyce indices for both BRT (Figure 8) and Maxent (Figure 9). This pattern is coherently observed for every species and every decade of model projection, with regression lines always displaying a positive slope. A threshold effect was consistent across these two species, indicating an improvement in model transferability as Schoener’s *D* increases, a feature that Maxent fails to exploit, likely due to differences in how each algorithm processes information and handles cross-correlation among explanatory variables.

Notably, BRT models outperformed Maxent. Only one model run produced negative Boyce values using BRT, whereas about half of the models generated with Maxent had Boyce index values lower than 0.5. Around 65% of the models showed high Boyce index values when Schoener’s *D* was higher than 0.5, a consistent finding recorded for every species and decade of model training. The high transferability observed for *I. ricinus* and *I. scapularis* using BRT is not observed with Maxent (Figure 9), a finding that we interpret as the inability of this algorithm to adapt to new combinations of climate conditions, project the model correctly, and produce a niche coherent with the environmental details gained from actual records of the projection decade.

Linear regression analyses revealed a statistically significant positive relationship between Schoener’s D and the Boyce Index across all species transfers (see Figure 8 and Figure 9). Higher historical niche overlap consistently yielded higher predictive performance in temporal projections, supporting the hypothesis that initial niche coverage strongly constraints model transferability over multi-decadal scales.

**Figure 10:**
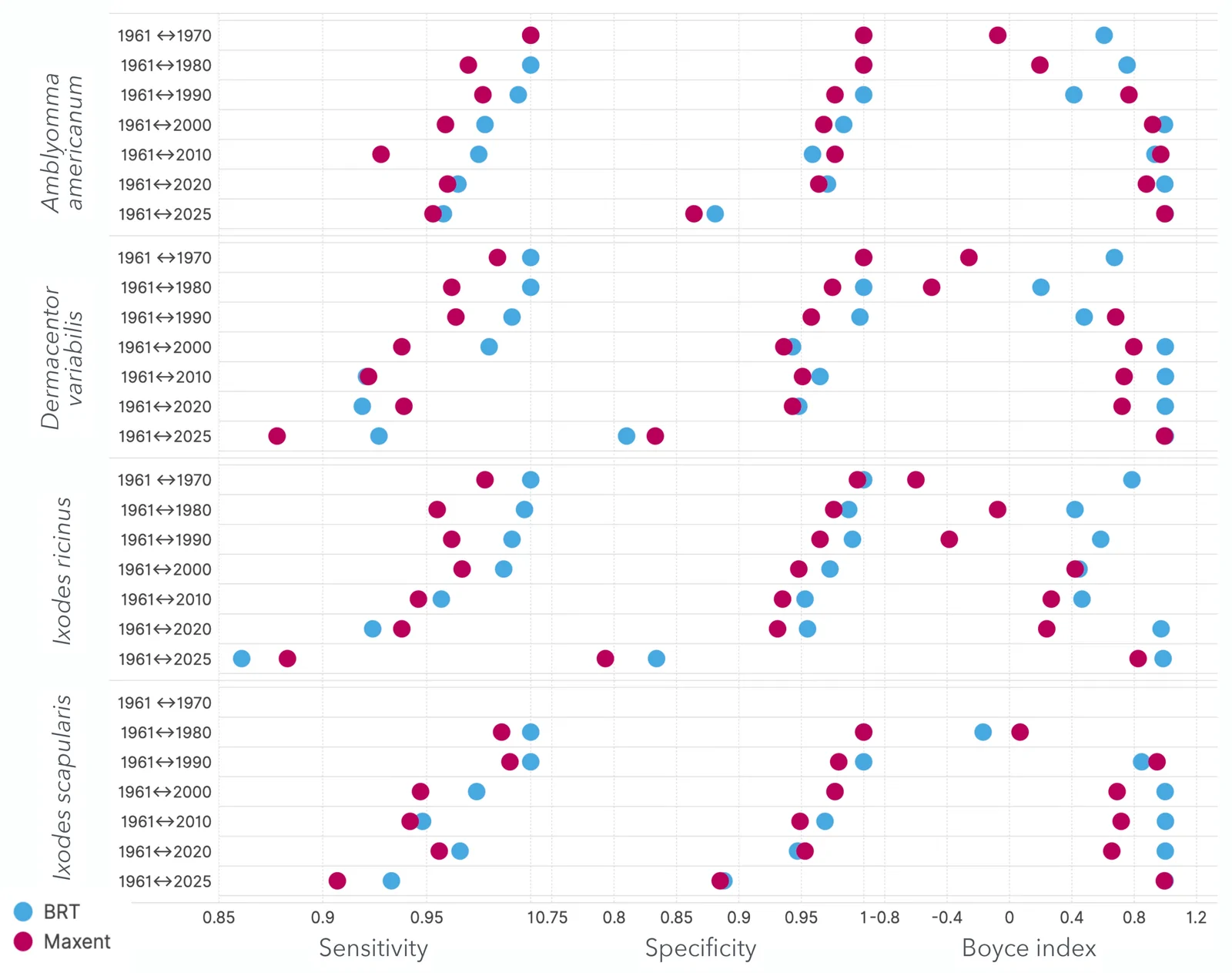
The relationships between the decades of training and projection, and the values of model sensitivity and specificity and the Boyce index. Colours mean for the algorithm used. The chart shows these indexes for models *accumulatively* trained with records and climate and then projected into 2021–2025. Labels at left of the chart, for each species, display the decades of training. A set of models was trained with the complete set of records and climate from 1961 to 2025, that, as expected, are the best models in both training and transferability.

Figure 10 summarizes the critical indices regarding training performance and model transferability using either BRT or Maxent. Sensitivity and specificity are lower for models trained with data spanning several decades, while the Boyce index is consistently high in these runs. Conversely, both sensitivity and specificity tend to be higher when models are trained with data from only one or a few decades, but the Boyce index then drops dramatically. While sensitivity and specificity explain how the model performed during training, the Boyce index explains the transferability rate. Models trained with limited data look unrealistically well-informed but critically fail when projected. On the other hand, models trained with as much information as possible (several decades) appear poorly performing during training but achieve the highest rates of transferability.

Collectively, our results reinforce the conclusion that tick distribution models built upon current georeferenced records and projected into future climates are inherently unreliable. This lack of robustness is driven by: (a) the methodological limitations of Maxent when handling highly collinear variables, and (b) a fundamental deficit in our baseline knowledge of actual tick distributions, which cannot be easily improved because it is impossible to determine when the entirety of the niche has been successfully captured and entered into the model. Distribution models regarding climate stability should be projected into future conditions only when the complete niche of each species is captured, something that remains virtually impossible to ascertain.

## 4. Discussion

Our study evaluated the reliability of projecting climate-driven species distribution models (SDMs) into future environmental conditions by taking advantage of a unique validation framework. Instead of comparing predictions against hypothetical future scenarios, we trained models using occurrence records from successive decades and projected them onto subsequent periods for which both climate and independent occurrence data were already available. This approach allowed a direct assessment of temporal transferability under realistic conditions. Across four widespread tick species and two modeling algorithms, our results consistently showed that models producing satisfactory calibration statistics frequently failed to reproduce the climatic niche represented by independent records from subsequent decades. We therefore argue that good model performance during calibration should not be interpreted as evidence of reliable temporal projections unless transferability is explicitly evaluated.

The results further indicate that this lack of robustness cannot be attributed exclusively to the modeling algorithm. Rather, it reflects a more fundamental limitation common to all correlational SDMs: the incomplete representation of the species’ climatic niche in the occurrence records used for model calibration [6,13,15]. Regardless of the algorithm employed, the model can only infer relationships from the portion of environmental space sampled by available observations. Environmental conditions that remain unsampled cannot contribute to model calibration and inevitably become sources of uncertainty when projections are transferred to different climatic combinations. Consequently, the reliability of future projections is constrained not only by model structure but also by the completeness with which present-day ecological information has been captured.

The reliability of occurrence datasets depends on survey intensity, geographical coverage, temporal continuity, taxonomic accuracy, and many additional sources of observational bias. These limitations affect all organisms but are especially important for medically relevant arthropods whose surveillance has increased dramatically only during the last decades. Consequently, the climatic niche represented by historical datasets may reflect patterns of scientific activity as much as the true environmental limits of the species.

Our results illustrate this problem clearly. Models trained with records from 1 decade generally predicted the immediately following decade better than more distant periods, yet transferability remained consistently lower than expected when projected into more “distant” climates. The progressive improvement through time in the last decades parallels the continuous accumulation of occurrence records and suggests that models gradually incorporate a more complete representation of the climatic niche rather than simply benefiting from increasing sample size.

This interpretation also provides an alternative perspective for recent reports of apparent distributional changes. For example, while the recent expansion of *Amblyomma americanum* is well documented [32], interpretations proposing an “ancestral range recovery” [33] should be considered cautiously because historical occurrence datasets may have captured only a limited fraction of the species’ environmental niche. Likewise, projections of future distributions are likely to become progressively more reliable as surveillance expands and larger fractions of the realised climatic niche become represented by georeferenced records [34]. The issue is therefore not whether climate change is modifying distributions, but whether historical datasets adequately describe the ecological space from which future projections are derived.

The extraordinary increase in tick surveillance during the last 3 decades reinforce this interpretation. Between 2020 and 2025 alone, 38%, 67%, 37%, and 42% of all currently available georeferenced records for *A. americanum*, *D. variabilis*, *I. ricinus,* and *I. scapularis*, respectively, were collected. Such figures illustrate that our present understanding of tick distributions is still evolving rapidly, and that models calibrated with older datasets inevitably represented only a fraction of the environmental niche currently known. This finding in our “simulated” future projections exemplifies clearly what could be the outcome when actual (current) data are projected into the uncertainty of climate scenarios.

The broader implication is that uncertainty in future SDM projections originates long before the projection step itself [35]. Predictive models ultimately depend on the quality and completeness of the occurrence data from which ecological relationships are inferred. No modeling algorithm, regardless of its statistical sophistication, can reconstruct portions of the climatic niche that were never represented in the calibration dataset [36,37,38].

One of the strengths of this study is that it allows the relative contribution of data availability and modeling methodology to be disentangled. Both Maxent and BRT produced comparable calibration statistics and similar contemporary predictions, yet their temporal transferability differed substantially. Although BRT generally outperformed Maxent, neither algorithm was able to overcome the limitations imposed by incomplete ecological information. This finding suggests that algorithm selection influences projection performance.

The poorer temporal performance of Maxent is nevertheless consistent with previous methodological studies that address the transferability of species distribution models [10,12,16,37,38]. Maxent remains one of the most widely used algorithms for ecological niche modeling because of its flexibility and generally excellent calibration performance [10,11,32]. However, future projections depend on the stability of the statistical relationships learned during model calibration [37]. When explanatory variables are highly correlated, these relationships may not remain constant through time. As climatic conditions evolve, historical patterns of collinearity progressively change, generating combinations of environmental variables that were absent during model training. Under these circumstances, models calibrated on historical correlations must extrapolate beyond the environmental relationships originally represented in the occurrence data, increasing projection uncertainty.

Our results are fully consistent with this interpretation. The marked changes observed in niche stability, expansion, and unfilling are difficult to explain solely by the magnitude of climatic change occurring between consecutive decades. Instead, they indicate that relatively small modifications in the relationships among explanatory variables are sufficient to alter the environmental space recognised by the model. A well-calibrated ecological model should preserve the biological relationships defining species suitability under changing climatic conditions. Conversely, when statistical correlations rather than ecological mechanisms dominate model calibration, projections become increasingly unstable as environmental combinations diverge from those represented in the training dataset.

This limitation also explains why standard procedures intended to reduce multicollinearity should be interpreted cautiously. Variable selection based on Variance Inflation Factors (VIF) undoubtedly improves statistical independence among predictors, but it may simultaneously remove variables carrying biologically meaningful information [13]. Likewise, replacing the original climatic variables by principal components effectively eliminates collinearity, yet it transforms ecologically interpretable climatic attributes into synthetic statistical axes. For organisms such as ticks, whose development and survival depend on the timing of seasonal climatic events rather than annual averages alone, these transformations inevitably reduce biological interpretability. Improved statistical performance should therefore not be confused with improved ecological realism.

The same argument applies to the widespread use of the Bioclim variables derived from WorldClim. These variables were developed as general descriptors of climate [25], not as mechanistic representations of the environmental processes regulating the biology of particular organisms. Their popularity has undoubtedly facilitated comparisons among studies, but their routine application has also encouraged the implicit assumption that annual or seasonal climatic summaries adequately represent the environmental drivers of species distributions [39]. For ticks, however, survival, development, and host-seeking activity are regulated by climatic conditions occurring during relatively short periods of the annual cycle [14]. Climatic averages may therefore obscure precisely those environmental thresholds that determine persistence or local extinction.

The harmonic representation of climatic seasonality adopted here was intended to address this limitation by preserving the temporal structure of climate while reducing dimensionality. Rather than describing climate through a collection of correlated annual summaries, harmonic coefficients retain information on average conditions, seasonal amplitude, and annual timing, variables that more closely reflect the environmental phenology experienced by the organism. Although our objective was not to compare alternative climatic datasets, the improved consistency obtained with these predictors supports the broader principle that explanatory variables should represent biologically meaningful processes rather than statistically convenient summaries.

An important finding is the strong relationship between Schoener’s *D* and the Boyce index observed across species, decades, and modeling algorithms. As the climatic niche represented in the calibration dataset becomes increasingly similar to that represented during the projection period, model transferability improves accordingly. This relationship demonstrates that predictive performance depends primarily on how completely the environmental niche has been characterised rather than on the absolute number of occurrence records available. Large datasets do not necessarily translate into robust projections if they repeatedly sample the same portion of environmental space, whereas comparatively fewer records may be highly informative when they encompass a broader range of ecological conditions.

From this perspective, environmental representativeness becomes more important than sample size itself. The consistently higher Boyce values observed when models were projected only 1 decade ahead indicate that the ecological knowledge accumulated over relatively short periods remains largely coherent, whereas projections across longer time intervals progressively diverge from the realised climatic niche represented by newly available records.

This distinction has important implications beyond tick ecology. Species distribution models are frequently evaluated according to statistical measures of calibration accuracy, yet their ultimate purpose is often to anticipate distributions under environmental conditions that have not yet occurred [35,36,37,38,40]. Our results demonstrate that these two objectives are not equivalent. A model may reproduce present-day observations with high apparent accuracy while remaining unable to predict future distributions if the environmental niche represented during calibration is incomplete. Consequently, confidence in long-term projections should derive not only from model fit, but also from explicit evidence that the sampled occurrence records adequately encompass the ecological space occupied by the species.

This limitation is unlikely ever to disappear completely. Because the true environmental niche of any species is unknown, there is no objective criterion by which researchers can determine whether available occurrence records have fully characterised its ecological tolerance. Every correlational SDM therefore represents, to some extent, an approximation of an unknown niche rather than the niche itself. Future projections should consequently be interpreted as hypotheses conditional upon the completeness of current ecological knowledge, rather than as definitive forecasts of future distributions.

Our findings therefore support a more cautious interpretation of climate-driven projections of tick distributions. Rather than questioning the usefulness of SDMs, they emphasise the need to distinguish between statistical prediction and ecological inference. Future models will undoubtedly benefit from improved algorithms, higher-resolution climate data, and increasingly sophisticated computational methods [41]. However, these methodological advances cannot compensate for ecological information that is absent from the calibration dataset. Improving surveillance, expanding the environmental representativeness of occurrence records, and selecting predictors that reflect biologically meaningful processes remain the most effective strategies for increasing the reliability of future projections.

Several limitations should be taken into consideration when interpreting our findings. First, environmental predictors were restricted to macroclimatic variables. Although vegetation structure and land cover are key micro-environmental determinants of tick survival, homogeneous, high-resolution historical land-use datasets spanning the 1961–1990 baseline are unavailable at regional scales, and using contemporary land cover would introduce severe temporal mismatch. Second, while historical data cleaning and temporal matching were strictly enforced, historical biodiversity records inherently suffer from uneven spatial sampling effort over time. Future studies combining long-term climatic baselines with fine-scale historical microhabitat reconstructions may further refine our understanding of niche dynamics.

In conclusion, the principal limitation of temporally transferred species distribution models does not lie in the projection algorithm itself, but in the incomplete representation of the realised climatic niche available during model calibration. Algorithmic improvements may reduce statistical error, but they cannot reconstruct ecological information that has never been observed. The reliability of future climate projections therefore depends fundamentally on the quality, completeness, and ecological representativeness of the occurrence records on which every model is built. This principle extends well beyond ticks and should be considered whenever species distribution models are used to forecast biological responses to environmental change.

## 5. Conclusions

Our study provides strong empirical evidence indicating that incomplete niche representation is a primary constraint on model performance in temporal projections of tick models. The evidence presented here substantiates and refines a critical premise: the incomplete mapping of current tick distributions is not merely a minor technical limitation, but a structural epistemological constraint that propagates directly into predictive modeling frameworks. The fundamental paradox of these tools is that their ability to anticipate future vector range shifts depends entirely on a comprehensive characterisation of present-day distributions, the very data that are most lacking and difficult to acquire.

Consequently, without a complete baseline of tick and tick-borne pathogen distributions, our capacity to accurately model future climate impacts will remain severely compromised. To move beyond these inherent flaws, future research must shift away from the uncritical application of automated, large-scale bioclimatic variables. Instead, priority must be given to establishing robust, systematically coordinated surveillance frameworks, employing high-resolution temporal climate series, and developing novel methodologies capable of accounting for both seasonal biology and the dynamic nature of ecological niches.

## Figures and Tables

**Table 1:** Number of records available for eight tick species, initially selected for the study. The species *Amblyomma variegatum*, *Dermacentor andersoni*, *Rhipicephalus annulatus*, and *Rhipicephalus microplus* were removed because the low number of records or the patterns of reporting, with decades in which georeferenced and dated records were unavailable.

Species	1901–1910	1911–1920	1921–1930	1931–1940	1941–1950	1951–1960	1961–1970	1971–1980	1981–1990	1991–2000	2001–2010	2011–2020	2021–2025	Total
***Amblyomma americanum***		5	8	38	87	43	50	82	111	912	2974	3866	5029	13,205
***Amblyomma variegatum***	104	30	43	79	88	389	1432	181	159	116	4	52	27	2704
***Dermacentor andersoni***		2	4	29	118	60	40	2	1	5	67	52		380
***Dermacentor variabilis***	7		9	79	179	121	88	134	203	316	767	3786	11,638	17,327
***Ixodes ricinus***	30	24	15	60	112	216	426	282	140	499	852	10,056	7495	20,213
***Ixodes scapularis***			2	17	36	16	33	79	166	913	2630	4939	6292	15,123
***Rhipicephalus annulatus***			8	6	21	33	249	188	11	56	1	3	14	590
***Rhipicephalus microplus***	3	5	9	15	231	199	321	15	97	14	2	9	19	939
**Total**	141	61	81	302	620	845	2069	760	780	2761	7294	22,751	30,481	68,952

## Data Availability

All the data generated in this study are available at https://figshare.com/s/dbe2d2e5d8aad1205ae8 (accessed on 13 August 2026). This material contains all the maps projected for this study, as well as all the indexes validating the models. The DOI of the resources is 10.6084/m9.figshare.33050654, which will be affective only after publishing.

## Abbreviations

The following abbreviations are used in this manuscript:

AUC	Area Under the Curve
TSS	True Skill Statistic
CBI	Continuous Boyce Index
PCA	Principal Components Analysis
SDM	Species Distribution Models
BRT	Boosted Regression Trees
VIF	Variance Inflation Factor
